# Further Evidence on Efficacy of Diet Supplementation with Fatty Acids in Ocular Pathologies: Insights from the EAE Model of Optic Neuritis

**DOI:** 10.3390/nu10101447

**Published:** 2018-10-06

**Authors:** Filippo Locri, Maurizio Cammalleri, Alessandro Pini, Massimo Dal Monte, Dario Rusciano, Paola Bagnoli

**Affiliations:** 1 Department of Biology, University of Pisa, via San Zeno 31, 56127 Pisa, Italy; filippo.locri1@gmail.com (F.L.); maurizio.cammalleri@unipi.it (M.C.); massimo.dalmonte@unipi.it (M.D.M.); 2 Interdepartmental Research Center Nutrafood ‘‘Nutraceuticals and Food for Health’’, University of Pisa, via del Borghetto 80, 56124 Pisa, Italy; 3 Department of Experimental and Clinical Medicine, University of Florence, Viale Pieraccini 6, 50139 Firenze, Italy; alessandro.pini@unifi.it; 4 Sooft Italia SpA, Contrada Molino 17, 63833 Montegiorgio (FM), Italy; dario.rusciano@sooft.it

**Keywords:** optic neuritis, myelin oligodendrocyte glycoprotein, neuroinflammation, macrophage polarization, demyelination, photopic ERG.

## Abstract

In the experimental autoimmune encephalomyelitis (EAE) mouse model of optic neuritis, we recently demonstrated that diet supplementation with a balanced mixture of fatty acids (FAs), including omega 3 and omega 6, efficiently limited inflammatory events in the retina and prevented retinal ganglion cell (RGC) death, although mechanisms underlying the efficacy of FAs were to be elucidated. Whether FAs effectiveness was accompanied by efficient rescue of demyelinating events in the optic nerve was also unresolved. Finally, the possibility that RGC rescue might result in ameliorated visual performance remained to be investigated. Here, the EAE model of optic neuritis was used to investigate mechanisms underlying the anti-inflammatory effects of FAs, including their potential efficacy on macrophage polarization. In addition, we determined how FAs-induced rescue of RGC degeneration was related to optic nerve histopathology by performing ultrastructural morphometric analysis with transmission electron microscopy. Finally, RGC rescue was correlated with visual performance by recording photopic electroretinogram, an efficient methodology to unravel the role of RGCs in the generation of electroretinographic waves. We conclude that the ameliorative effects of FAs were dependent on a predominant anti-inflammatory action including a role on promoting the shift of macrophages from the inflammatory M1 phenotype towards the anti-inflammatory M2 phenotype. This would finally result in restored optic nerve histopathology and ameliorated visual performance. These findings can now offer new perspectives for implementing our knowledge on the effectiveness of diet supplementation in counteracting optic neuritis and suggest the importance of FAs as possible adjuvants in therapies against inflammatory diseases of the eye.

## 1. Introduction

The recent trend in ocular pathologies is to use medicinal natural products, which may offer the same efficacy of conventional drugs without so many side effects. In particular, nutritional approaches may contribute to counteract eye diseases with a major inflammatory component as an increasing amount of scientific data highlights the ability of specific nutrients to cross the blood retinal barrier and to modulate inflammatory pathways that account for eye diseases [1]. Among diet supplements, fatty acids (FAs) including omega 3 and omega 6, are extensively investigated in terms of prophylactic benefits, potential harms, and optimal use in ocular pathologies, but their benefits are often controversial. For instance, the Age-Related Disease Study 2 (AREDS2) seems to exclude preventive effects of omega-3 FAs against dry age-related macular degeneration (AMD), although disregarding a role of FAs in AMD progression should be reconsidered [2]. Indeed, in a murine model of dry AMD [3], we recently demonstrated that diet supplementation with a calibrated mixture of the long chain omega 3 and omega 6 FAs drastically ameliorates complement-mediated effects, thus decreasing macrophage recruitment and the production of pro-inflammatory and angiogenic cytokines, which in turn improve degenerative events in the retina and the choroid [4]. In addition, using the experimental autoimmune encephalomyelitis (EAE) mouse model of optic neuritis, we found that daily gavage with a FAs mixture delays the onset of EAE and prevents, at least in part, the death of retinal ganglion cells (RGCs) through a major anti-inflammatory action [5]. This has been shown to occur without affecting the infiltration of macrophages in the retina thus eventually preserving their correct release of trophic and growth factors that are known to prevent optic nerve degeneration [6]. Ameliorative effects of FAs are in line with previous findings in the EAE model demonstrating that a combination of FAs, antioxidants, free radicals, and amino acids protects the optic nerve from demyelination and promotes the formation of the myelin sheath [7].

Understanding the mechanisms underlying the anti-inflammatory action of FAs can offer new perspectives not only for implementing our knowledge on the effectiveness of diet supplements in optic neuritis, but also for expanding a complementary nutritional intervention in multiple sclerosis (MS). In addition, whether FAs efficacy is accompanied by a rescue of demyelinating events in the optic nerve remains to be clarified. Finally, the possibility of correlating RGC rescue with improved visual performance may add further value to the use of FAs since a few neuroprotectants have been shown to successfully rescue RGC function [8,9,10]. In the present study, the EAE model of optic neuritis was used to clarify mechanisms underlying the anti-inflammatory effects of FAs including an investigation of the possibility that FAs may shift macrophage polarization from the M1 inflammatory phenotype towards the anti-inflammatory M2 phenotype [11]. To this aim, in retinal homogenates, we evaluated the effect of FAs on (i) the transcript level of M1-and M2-related markers, (ii) transcription factors related to M1/M2 activation, and (iii) interleukin (IL)-10 and Arginase-1 (Arg-1) that are well-established markers of the M2 phenotype [11]. In addition, we determined how FAs-induced rescue of RGC death may be related to ameliorated optic nerve histopathology by performing ultrastructural morphometric analysis by transmission electron microscopy. Finally, we aimed at correlating RGC rescue with visual function by determining the effect of FAs on the photopic negative response (PhNR) of an electroretinogram (ERG), an efficient tool to unravel the role of RGCs in the generation of electroretinographic waves [12].

## 2. Materials and Methods 

### 2.1. Animals 

Experiments were carried out in accordance with the recommendations in the Guide for the Care and Use of Laboratory Animals of the National Institutes of Health, the ARVO Statement for the Use of Animals in Ophthalmic and Vision Research, the Italian guidelines for animal care (DL 26/14), and the European Communities Council Directive (2010/63/UE). The experimental procedures were approved by the Commission for Animal Wellbeing of the University of Pisa (Permit Number: 0009069/2014). All efforts were made to reduce animal suffering and the number of animals. C57BL/6J mice were purchased from Charles River Laboratories Italy (Calco, Italy), mated in-house to a breeding colony in a regulated environment (23 ± 1 °C, 50 ± 5% humidity) with a 12 h light/dark cycle (lights on at 08:00 a.m.) and provided with a standard diet and water ad libitum. In the present study, 36 females (8 week-old) were used as more susceptible than males to develop MS [13]. Indeed, MS as many other diseases with an autoimmune etiology have a skewed sex distribution with females being affected more frequently than males presumably due to hormonal and gender influences on the immune response, as well as to genetic influences [14]. On the other hand, the susceptibility to develop experimental MS linked to sex differences has been long debated in the mouse model [15]. In addition, sex restriction in animal studies should be taken cautiously to avoid potential pitfalls as indicated in the European Commission funding program Horizon 2020.

### 2.2. Induction of EAE 

As described previously, mice were anesthetized by isoflurane and 18 received subcutaneous injections of 200 μg myelin oligodendrocyte glycoprotein (MOG) peptide (MOG_35–55_; Anaspec, Freemont, CA, USA) emulsified in Complete Freund’s Adjuvant (CFA; Sigma-Aldrich, St. Louis, MO, USA), containing 2.5 mg/mL *Mycobacterium tuberculosis*. Eighteen mice (from now on referred as controls) were injected with an equal volume of phosphate-buffered saline (PBS) and CFA. All animals received 200 ng pertussis toxin (Sigma-Aldrich, St Louis, MO, USA) in 0.1 mL PBS by intraperitoneal injection at 0 and 48 h post immunization. The EAE model of MS in the C57BL/6J mouse strain approximates the human disease although no peculiar susceptibility of this strain has been demonstrated. As determined by clinical EAE scores, C57BL/6J mice treated with MOG were found to develop an ascending paralysis that was either monophasic or chronically nonremitting with first symptoms after 9 to 14 days, disease maximum approximately 3 to 5 days after disease onset and partial symptom recovery over the next 10 to 20 days [16]. Clinical EAE scores were graded blindly and daily using an established standard scoring system [17]. Nine MOG-treated mice, which were sacrificed at the day 16 postimmunization corresponding to the score 2 ± 0.5 when optic neuritis becomes well established [18], were compared with nine control mice. Six mice for each group were used for electroretinography before sacrifice.

### 2.3. Dietary Supplementation 

In line with a previous study [5], 9 MOG-treated mice and nine controls received by oral gavage (starting from the day of immunization until the day 16 when the animals were sacrificed) a mixture of saturated and unsaturated FAs including 20% of saturated FAs, while 17.5% of unsaturated FAs. Of this mixture, 3.75 mg suspended in 200 μL of 10% sucrose in water were administered to the animals. The mixture composition was described previously [5].

### 2.4. Isolation of Total RNA and Proteins

Mice were deeply anesthetized and euthanized by cervical dislocation. Retinas were rapidly dissected and stored at −80 °C until use. For each analysis, nine independent samples, each containing two retinas from two mice per experimental condition were used. Total RNA and proteins were extracted using the AllPrep RNA/Protein Kit (Qiagen, Valencia, CA, USA) according to the manufacturer instructions.

### 2.5. Quantitative Real Time PCR 

First-strand cDNA was generated from 1 μg of total RNA (QuantiTect Reverse Transcription Kit, Qiagen, Valencia, CA, USA). Real-time PCR amplification was performed with SsoAdvanced Universal SYBR Green Supermix (Bio-Rad Laboratories, Hercules, CA, USA) on a CFX Connect Real-Time PCR detection system and software CFX manager (Bio-Rad Laboratories, Hercules, CA, USA). qPCR primer sets for C-X-C motif chemokine (CXCL)-10, CXCL-11, IL-12, IL-23, C-C motif chemokine (CCL)-2, CCL-22, cluster of differentiation-163 (CD-163), and Arg-1 were chosen to hybridize to unique regions of the appropriate gene sequence (see Table S1 for a complete list of assayed genes and primers). Amplification efficiency was near 100% for each primer pair (Opticon Monitor 3 software, Bio-Rad Laboratories, Hercules, CA, USA). Target genes were assayed concurrently with Rpl13a: a gene encoding ribosomal protein L13A. Samples were compared using the relative threshold cycle (Ct Method). The increase or decrease (fold change) was determined relative to control mice after normalization to Rpl13a. All reactions were performed in triplicate. 

### 2.6. Western Blot 

Samples containing 30 μg of proteins were subjected to SDS-PAGE, and β-actin was used as loading control. Gels were transblotted onto a PVDF membrane, and the blots were blocked in 3% skim-milk for 1 h at room temperature, followed by incubation overnight at 4 °C with antibodies listed in Table S2. Blots were incubated for one hour at room temperature with HRP-conjugated secondary antibodies (1:5000) and developed with Clarity Western enhanced chemiluminescence substrate (Bio-Rad Laboratories, Inc., Hercules, CA, USA), images were acquired (ChemiDoc XRS+; Bio-Rad Laboratories, Inc., Hercules, CA, USA), and the optical density (OD) of the bands was evaluated (Image Lab 3.0 software; Bio-Rad Laboratories, Inc., Hercules, CA, USA). The data were normalized to the corresponding OD of β-actin, the signal transducer and activator of transcription 3 (STAT3) or the nuclear factor kappa-light-chain-enhancer of activated B cells (NF-κB) as appropriate. All experiments were performed in duplicate. 

### 2.7. Electron Microscopy and Quantitative Analysis

After extraction, optic nerves were fixed by immersion in Karnofsky’s solution and stored in the fixative solution for 3 days at 4 °C. Samples were rinsed in 0.1 M cacodylate buffer, postfixed in 1% osmium tetroxide, dehydrated in ascending dilution series of acetone, and then embedded in Epon 812. Semithin sections were cut to evaluate quality and orientation of the tissue. Ultrathin cross-sections of the optic nerves were prepared and mounted on grids, stained with UranyLess TEM staining, and examined under a JEM 1010 electron microscope (Jeol, Tokyo, Japan) at 20 kV and 50 kV. Images were captured using a CCD digital camera. For analysis of axon myelinated proportions [19], 10 regions of interest (ROIs), randomly taken in the subcentral and subperipheral areas of the four optic nerve quadrants (nasal, temporal, dorsal, or ventral) for each sample (five optic nerves for each experimental group), were imported into NIH Fiji and all axons within the ROIs were quantified as myelinated or nonmyelinated. The *g*-ratio is defined as the ratio of the inner axonal diameter to the total outer diameter; is a highly reliable ratio for assessing axonal myelination [20]. It was calculated using digitalized images that were imported into NIH Fiji. For each animal, 30–50 myelinated axons were measured. 

### 2.8. Electroretinography

Full-field ERG was recorded using a Ganzfeld stimulator (Biomedica Mangoni, Pisa, Italy). After overnight dark adaptation, mice were prepared for recording under dim red light and anesthetized by intraperitoneal injection of Avertin. Pupils were dilated with 0.5% atropine, the cornea was intermittently irrigated with saline solution to prevent clouding of the ocular media, and a heating pad was used to keep the body temperature at 37 °C. The ERG responses were recorded through silver/silver chloride corneal electrodes and a forehead reference electrode, and a ground electrode was placed on the tail. Photopic, cone-mediated responses were recorded following 10-min light adaptation on the background light intensity of 30 cd/m^2^. Recordings were obtained at the light intensity of 3 cd-s/m^2^. From each animal, 10 waveforms were recorded and the values were averaged. The Photopic Negative Response (PhNR) was identified as the first negative deflection after the b-wave and its amplitude was calculated relative to baseline (0 μV). The values of PhNR amplitudes were compared among the groups.

### 2.9. Statistical Analysis 

All data were analyzed by the Shapiro–Wilk test to certify normal distribution. Statistical significance was evaluated with Prism 5.03 (GraphPad software, San Diego, CA, USA) using one-way analysis of variance (ANOVA) followed by Newman Keuls’ multiple comparison post-test or two-way ANOVA followed by Bonferroni’s multiple comparison post-test as appropriate. After statistical analysis, the data from different experiments were plotted and averaged in the same graph. Results were expressed as the mean ± standard error of the mean S.E.M. of the indicated n values. Differences with *p* < 0.05 were considered significant. 

## 3. Results

### 3.1. Supplementation with FAs Shifts M1 Macrophages toward M2 Phenotype

**Figure 1:**
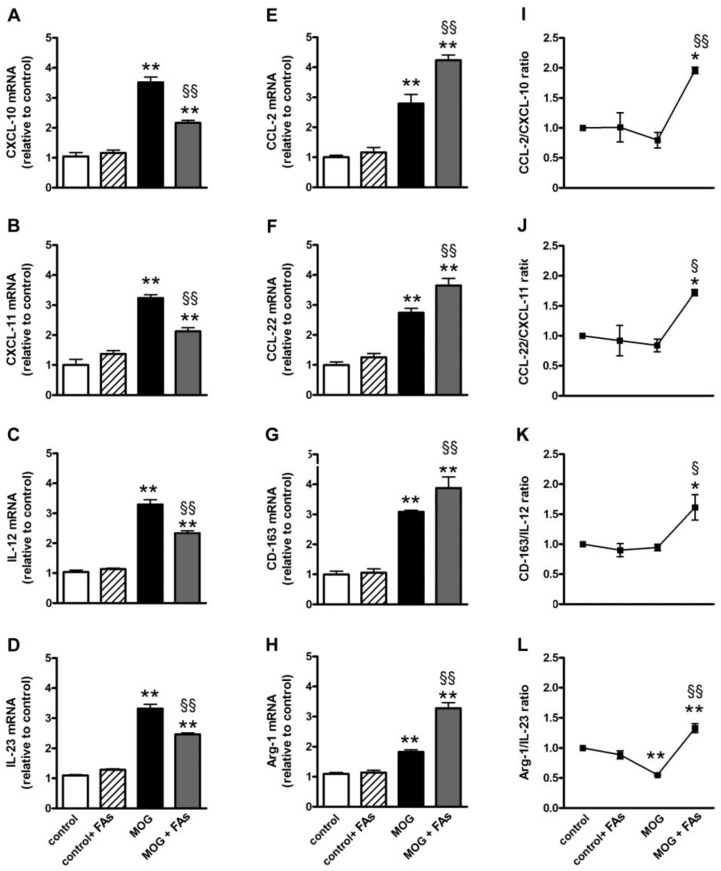
Dietary supplementation with fatty acids (FAs) reduces upregulated levels of M1-related markers including C-X-C motif chemokine (CXCL)-10 (in **A**), CXCL-11 (in **B**), interleukin (IL)-12 (in **C**), and IL-23 (in **D**), while additionally increases upregulated levels of M2-related markers including C-C motif chemokine (CCL)-2 (in **E**), CCL-22 (in **F**), cluster of differentiation-163 (CD-163, in **G**), and arginase-1 (Arg-1, in **H**). Transcript levels were evaluated in retinal homogenates from control and oligodendrocyte glycoprotein (MOG)-treated mice, without or with FAs supplementation by relative quantification with quantitative real-time PCR (qPCR). Data were analyzed by the formula 2^−ΔΔCT^ using ribosomal protein L13A (Rpl13a) as the internal standard. Ratio of the mean values of CCL-2 to CXCL-10 (**I**); CCL-22 to CXCL-11 (**J**); CD-163 to IL-12 (**K**); and Arg-1 to IL-23 (**L**). Data are shown as the mean ± S.E.M. (*n* = 9 for each experimental group). * *p* < 0.01; ** *p* < 0.001 versus control. ^§^ *p* < 0.01; ^§§^ *p* < 0.001 versus MOG (One way ANOVA followed by the Newman–Keuls multiple comparison post-hoc test). White bars, control mice; dashed bars, control mice with FAs supplementation; black bars, MOG-treated mice; grey bars, MOG-treated mice with FAs supplementation.

We investigated whether FAs could affect macrophage polarization by evaluating the transcript levels of M1- or M2-related markers in retinal homogenates. Indeed, macrophages are polarized toward an M1 phenotype in response to pro-inflammatory stimuli, whereas M2 macrophages display anti-inflammatory characteristics as IL-10 secretion or upregulated levels of the scavenger receptor CD-163 [11]. In respect to controls, MOG significantly increased both M1-related markers including CXCL-10, CXCL-11, IL-12, and IL-23 (Figure 1A–D) and M2-related markers including CCL-2, CCL-22, CD-163, and Arg-1 (Figure 1E–H). In particular, CXCL-10 (Figure 1A), CXCL-11 (Figure 1B), IL-12 (Figure 1C), IL-23 (Figure 1D), CCL-2 (Figure 1E), CCL-22 (Figure 1F), CD-163 (Figure 1G), and Arg-1 (Figure 1H) were increased by 3.3-, 3.2-, 2.8-, 2.5-, 2.8-, 2.7-, 3.0-, and 2.0-fold (*p* < 0.001). Supplementation with FAs did not affect the levels of these markers in controls. In MOG-treated mice, upregulated levels of M1-related markers were significantly decreased by FAs, while additional increase of M2-related markers was determined after FAs supplementation. In particular, FAs reduced levels of CXCL-10, CXCL-11, IL-12, and IL-23 by 1.6-, 1.5-, 1.4-, and 1.4-fold (*p* < 0.001), whereas they increased levels of CCL-2, CCL-22, CD-163, and Arg-1 by 1.5-, 1.3-, 1.3-, and 1.8-fold (*p* < 0.001). To evaluate a possible predominance of M2 over M1 macrophages, we analyzed the ratio of the mean values of M2 to M1. Ratio values of >1 indicated M2 predominance, while ratio values of <1 indicated M1 predominance [21,22]. The mRNA ratio in controls was standardized at 1. As shown in Figure 1I–L, after supplementation with FAs, for all markers analyzed, the ratio of M2 to M1 was >1 suggesting an M2 predominance.

**Figure 2:**
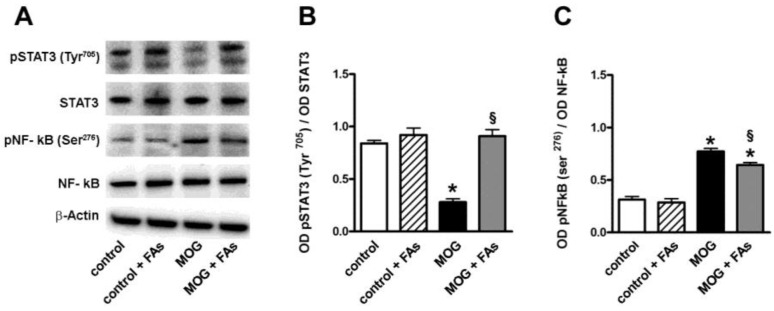
Dietary supplementation with FAs prevents MOG-induced downregulation of signal transducer and activator of transcription 3 (STAT3) phosphorylation and reduces upregulated levels of phosphorylated nuclear factor kappa-light-chain-enhancer of activated B cells (NF-κB). (**A**) Representative blots from retinal homogenates of control and MOG-treated mice, with or without FAs supplementation. Protein levels of pSTAT3 (Tyr^205^) (**B**) and pNF-κB (Ser^276^) (**C**) were evaluated by Western blot using STAT3 or NF-κB as loading controls. Data from densitometric analysis are shown as the mean ± S.E.M. (*n* = 9 for each experimental group). * *p* < 0.001 versus control. ^§^ *p* < 0.001 versus MOG (One way ANOVA followed by the Newman–Keuls multiple comparison post-hoc test). White bars, control mice; dashed bars, control mice with FAs supplementation; black bars, MOG-treated mice; grey bars, MOG-treated mice with FAs supplementation.

Western blotting experiments were also performed in order to evaluate the effect of FAs administration on transcription factors related to M1/M2 transition. As shown in Figure 2, in respect to controls, MOG decreased by 3.1-fold (*p* < 0.001) the phosphorylation of STAT3 at Tyr^705^, an important M2-related transcription factor [23], while increased by 2.8-fold (*p* < 0.001) the phosphorylation of NF-κB at Ser^276^, a key transcription factor related to macrophage M1 activation [24]. FAs supplementation did not affect the levels of these factors in controls, whereas, in MOG-treated mice, FAs increased the phosphorylation of STAT3 by 3.6-fold (*p* < 0.001), while decreased the phosphorylation of NF-κB by 1.4-fold (*p* < 0.001). 

**Figure 3:**
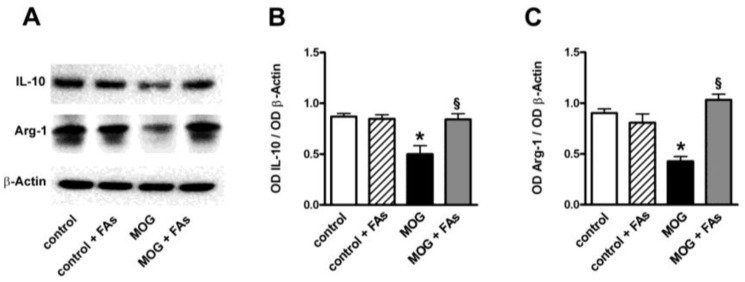
Dietary supplementation with FAs prevents MOG-induced downregulation of both IL-10 and Arg-1. (**A**) Representative blots from retinal homogenates of control and MOG-treated mice, with or without FAs supplementation. Protein levels of IL-10 (**B**) and Arg-1 (**C**) were evaluated by Western blot using β-actin as loading control. Data from densitometric analysis are shown as the mean ± S.E.M. (*n* = 9 for each experimental group). * *p* < 0.001 versus control. ^§^ *p* < 0.001 versus MOG (One way ANOVA followed by the Newman–Keuls multiple comparison post-hoc test). White bars, control mice; dashed bars, control mice with FAs supplementation; black bars, MOG-treated mice; grey bars, MOG-treated mice with FAs supplementation.

In additional experiments, we determined whether FAs might affect the levels of IL-10 and Arg-1, both well-established markers of M2 phenotypes [11]. As shown in Figure 3, MOG decreased both IL-10 and Arg-1 proteins by 1.9- and 2.1-fold (*p* < 0.001). FAs supplementation did not affect the levels of these markers in controls. In MOG-treated mice, FAs significantly increased the levels of both IL-10 (1.9-fold, *p* < 0.001) and Arg-1 (2.4-fold, *p* < 0.001). 

### 3.2. Supplementation with FAs Counteracts Optic Nerve Damage

**Figure 4:**
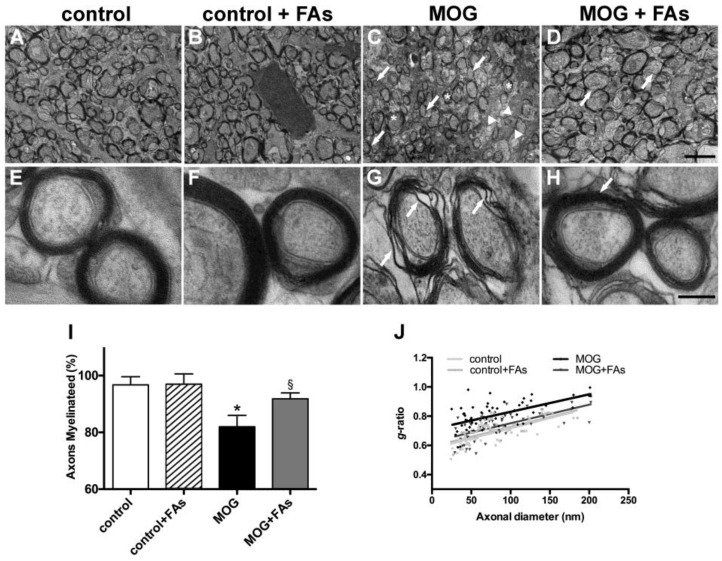
Dietary supplementation with FAs counteracts MOG-induced optic nerve demyelination. Representative electron micrographs of the optic nerves in control mice without (**A**) or with (**B**) FAs supplementation, MOG-treated mice (**C**), and MOG-treated mice with FAs supplementation (**D**). Calibration bar 2 µm. Higher-magnification micrographs are shown in (**E**) (control mice), (**F**) (control mice with FAs supplementation), (**G**) (MOG-treated mice), and (**H**) (MOG-treated mice with FAs supplementation). Calibration bar, 200 nm. (**I**) Quantification of myelinated axons. Data are shown as the mean ± S.E.M. (*n* = 5 for each experimental group) * *p* < 0.001 versus control, ^§^ *p* < 0.01 versus MOG (One way ANOVA followed by the Newman–Keuls multiple comparison post-hoc test). White bars, control mice; black bars, MOG-treated mice; grey bars, MOG-treated mice with FAs supplementation. (**J**) Analysis of mean *g*-ratio, the ratio of the inner axonal diameter to the total outer diameter (two-way ANOVA followed by Bonferroni’s multiple comparison post-test).

We studied the optic nerve of EAE animals using transmission electron microscopy and analyzed the degree of demyelination, and the putative myelin-protecting effect of FAs. As compared to control mice without or with FAs supplementation (Figure 4A,B), in MOG-treated mice, the optic nerve revealed evident demyelinating processes with nonmyelinated axons (arrows), thinly myelinated axons (arrowheads), and additional signs of myelin changes including delamination (asterisks) (Figure 4C). In MOG-treated mice with FAs supplementation, optic nerve myelination appeared almost comparable to that in controls with normally myelinated fibers, but also apparent myelinolysis and scattered thinly myelinated axons (arrows in Figure 4D). Higher-magnification images in control mice without or with FAs supplementation show myelinated axons of round-ovoid sectional profiles with microtubules and neurofilaments dispersed into the axoplasm. Surrounding the axons, myelin was found to remain compact with a regular periodicity without intramyelinic lacunae or vacuoles (Figure 4E,F). In MOG-treated mice without FAs supplementation, fibers revealed evident myelin changes including high levels of lamellar separation and widening (arrows in Figure 4G). In MOG-treated mice with FAs supplementation, fibers appeared round-ovoid shaped and showed almost compact myelin with a regular periodicity (arrow in Figure 4H). Morphometric analysis of the optic nerve fibers revealed a significant reduction of the number of myelinated fibers in MOG-treated mice as compared to controls without or with FAs supplementation (*p* < 0.001; Figure 4I). FAs administration prevented the demyelinating processes in the optic nerve and almost restored the number of myelinated fibers to that in control mice. As shown in Figure 4J, FAs supplementation to control mice did not affect the mean *g*-ratio of the myelinated axons whereas a significant increase could be observed in MOG-treated mice as compared to controls (0.83 ± 0.02 versus 0.66 ± 0.01, *p* < 0.01). The mean *g*-ratio was significantly lower in MOG-treated mice with FAs supplementation (0.71 ± 0.1; *p* < 0.05).

### 3.3. Supplementation with FAs Prevents the Reduction of PhNR Amplitude 

**Figure 5:**
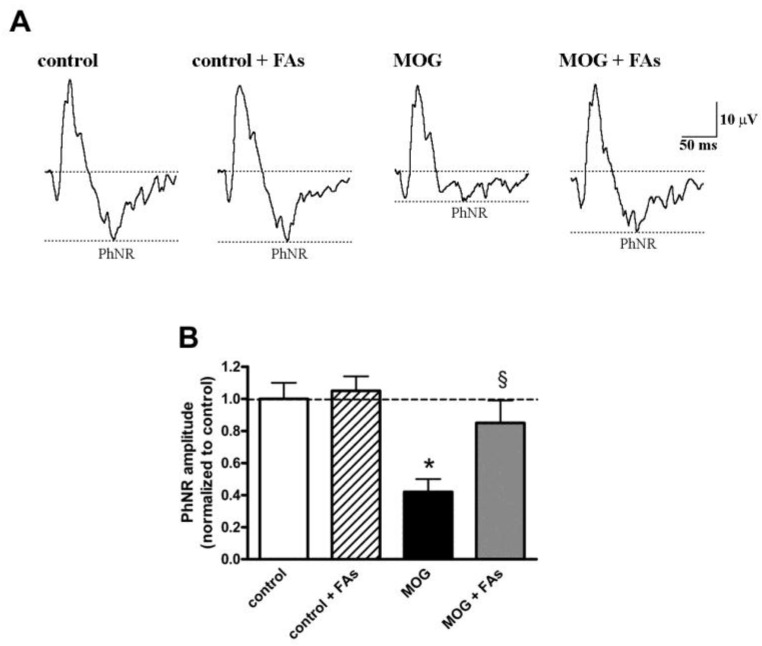
Dietary supplementation with FAs almost prevents MOG-induced reduction of photopic negative response (PhNR) amplitude. (**A**) Representative recordings in control and MOG-treated mice with or without FAs supplementation. (**B**) PhNR amplitudes were normalized to the amplitude of the control group. Data are shown as the mean ± S.E.M. (*n* = 6 for each experimental group). * *p* < 0.001 versus control. ^§^ *p* < 0.001 versus MOG (One way ANOVA followed by the Newman–Keuls multiple comparison post-hoc test). White bars, control mice; dashed bars, control mice with FAs supplementation; black bars, MOG-treated mice; grey bars, MOG-treated mice with FAs supplementation.

We asked the question of whether the efficacy of FAs on RGC survival was associated with ameliorated retinal function by analyzing the amplitude of the PhNR, a sensitive marker of the inner retinal function [25,26,27]. PhNR amplitude is altered in several retinal pathologies involving RGCs and including, among others, glaucoma, diabetic optic nerve atrophy, and optic neuritis [12]. The amplitude of the PhNR is proportional to the number of functional RGCs [28,29,30,31,32]. As shown in Figure 5, the amplitude of the PhNR was significantly reduced in MOG-treated mice by approximately 60% as compared to control mice (*p* < 0.01). FAs administration almost prevented the reduction in PhNR amplitude.

## 4. Discussion

Diet supplements enriched in omega-3 FAs are today available on the market and are used in patients with MS because of their possible role in restoring MS-associated inflammatory processes [33]. Although information in models of optic neuritis are scarce, neuroprotective effects of omega-3 FAs on RGC degeneration have been demonstrated in animal models of optic nerve crush [34] and results in the EAE model are limited to the demonstration of ameliorative effects of FAs on optic nerve inflammation [7,35,36]. On the other hand, the C57BL/6 mouse model of MOG-induced EAE although extensively used as a model of MS does not mimic the human disease. Indeed, approximately 85% of patients with MS display a relapsing-remitting disease that is not mirrored by the EAE model [37] although diet supplements enriched in omega-3 FAs have been positively used in MS patients [33].

As shown by our previous and present results, diet supplementation with FAs efficiently counteracts the effects of MOG when its administration starts the day of immunization. This protocol is in line with previous studies in which diet supplements are given after the pathology has been induced by MOG [5] although, the possibility exists that the incorporation of FAs into the diet before MOG-induction may be viewed as a preventive measure. Indeed, recent epidemiologic studies have reported a decreased incidence of MS among individuals with high intake of PUFAs [38,39,40] although conflicting results on a possible relationship between PUFA intake and decreased MS risk are also reported [41]. 

Our recent findings that in the EAE model, dietary supplementation with a mixture including omega 3 and omega 6 FAs results in neuroprotective effects on RGCs through a major anti-inflammatory action [5] opens an interesting field of investigation aimed at implementing the use of FAs as possible adjuvants in therapies against inflammatory diseases of the eye. In this respect, the fact that FAs drastically reduce inflammatory events in the retina, but do not affect its macrophage infiltration, suggests the possibility that they may indeed shift the M1 phenotype towards the M2 without intervening on the release of trophic factors that play an important role in counteracting optic nerve degeneration [6].

It is hypothesized that the transition from the pro-inflammatory M1 phenotype to the regulatory or anti-inflammatory M2 phenotype can lead to tissue repair and restoration of improved functional outcome [11]. As demonstrated here, FAs act in the retina by modulating the phenotype of macrophages, decreasing their pro-inflammatory activity and proportionally increasing their tissue restoring capabilities with a positive effect on neuronal rescue in line with finding that the M1/M2 phenotype balance plays an important role in optic neuritis progression [42]. Of the M1-related markers, IL-12 and IL-23 play a critical role in EAE development by inducing the release of CXCL-10 and CXCL-11 [43] that are both involved in macrophage recruitment [44]. Among M2-related markers, CCL-2 promotes axon regeneration in a rat spinal cord injury model [45], whereas CCL-22 contributes to the recruitment of T cells with anti-inflammatory function in the EAE model [46]. The fact that FAs supplementation shifts the macrophage balance towards the M2 phenotype is in line with the finding that in a rat model of optic neuropathy, supplementation with omega-3 FAs decreases RGC loss by regulating macrophage phenotype [47]. 

Additional evidence that FAs may act by promoting the shift towards the M2 phenotype originates from the demonstration that FAs are shown here to increase the levels of transcription factors related to M2 activation. Indeed, transcriptional regulation is central to the differential speciation of macrophages, and several major pathways have been described as essential for subset differentiation. Among them, STAT3 is the key transcription regulator of IL-10, a major anti-inflammatory mediator [48]. In particular, IL-10 binds the IL-10 receptor complex thus resulting in JAK1-mediated activation of STAT3 and repression of proinflammatory cytokines [49]. NF-kB, in contrast, orchestrates the expression of many inflammatory genes in response to various physiological and environmental stimuli [50]. As shown here, MOG decreases the phosphorylation of STAT3, while increasing the phosphorylation of NF-κB in line with previous studies demonstrating that in the EAE model, reduced activity of STAT3 increases the disease severity that is, in contrast, drastically ameliorated by NF-κB inactivation [51]. As also shown by the present results, supplementation with FAs almost reinstates control levels of pSTAT3, while its effects on pNF-kB appear less pronounced albeit significant. In this line, in rats with experimental brain injury, omega-3 FAs have been shown to inhibit NF-kB thus attenuating microglia-induced inflammation [52]. As a further evidence that FAs play a key role on macrophage polarization, we found that diet supplementation with FAs prevents MOG-induced downregulation of IL-10 and Arg-1; both of which are well-established markers of M2 phenotype and are both associated with attenuation of inflammation [11]. In particular, IL-10 is an important mediator of inflammation resolution through a major repression of proinflammatory cytokines [53], while Arg-1 promotes cytokine production thus contributing to resolution of inflammation and tissue repairing [54,55].

Overall, our results confirm the critical role of macrophages in optic neuritis and demonstrate for the first time that M2 macrophages become predominant after diet supplementation with FAs although further investigations are required to understand the mechanisms underlying the macrophage shift. In this respect, targeting M2 macrophages and their signaling pathways may be the key to discovering new avenues for the treatment of optic neuritis.

The anti-inflammatory action of FAs results in RGC rescue as demonstrated previously [5], and is concomitant with reduced optic nerve damage as observed here. Indeed, acute EAE is characterized by inflammation-dependent optic nerve damage [56] and suppression of inflammatory processes has been shown to protect the optic nerve from axonal demyelination [57]. Our finding that FAs supplementation exerts a myelin-protective role in the optic nerve is in line with previous results demonstrating that a combination of FAs, antioxidants, free radicals, and amino acids can effectively decrease axon demyelination in the optic nerve or in the spinal cord [7].

Finally, our finding that FAs may counteract, at least in part, RGC dysfunction adds the most conclusive evidence to the their use against optic neuritis. In fact, restored visual function is an important sign of neuroprotective efficacy of diet supplements and only a few neuroprotectants with antioxidant or anti-inflammatory activity seem to promote RGC survival with restoration of visual function in models of ocular pathologies [8,9,10]. As shown here, MOG-treated mice are characterized by reduced amplitude of the PhNR, which is almost prevented by FAs supplementation. The PhNR is a negative-going wave following the b-wave of the cone response, it originates in the inner retinal layer, and its amplitude is well-correlated with the thickness of the RGC layer, thus providing a direct, objective assessment of the changes in RGC function with the consequent clinical applications [25,26,27,29,58]. PhNR advantage is that can be recorded noninvasively using conventional ERG together with the a- and b-waves thus enabling the function of middle and outer retinal layers to be evaluated at the same time.

## 5. Conclusions

Taken together, the present evidence that FAs successfully ameliorate RGC dysfunction by acting as a major anti-inflammatory compound can now offer new perspectives not only for implementing our knowledge on the effectiveness of diet supplement in counteracting optic neuritis, but also for eventually expanding a complementary nutritional intervention in MS.

**Figure 6:**
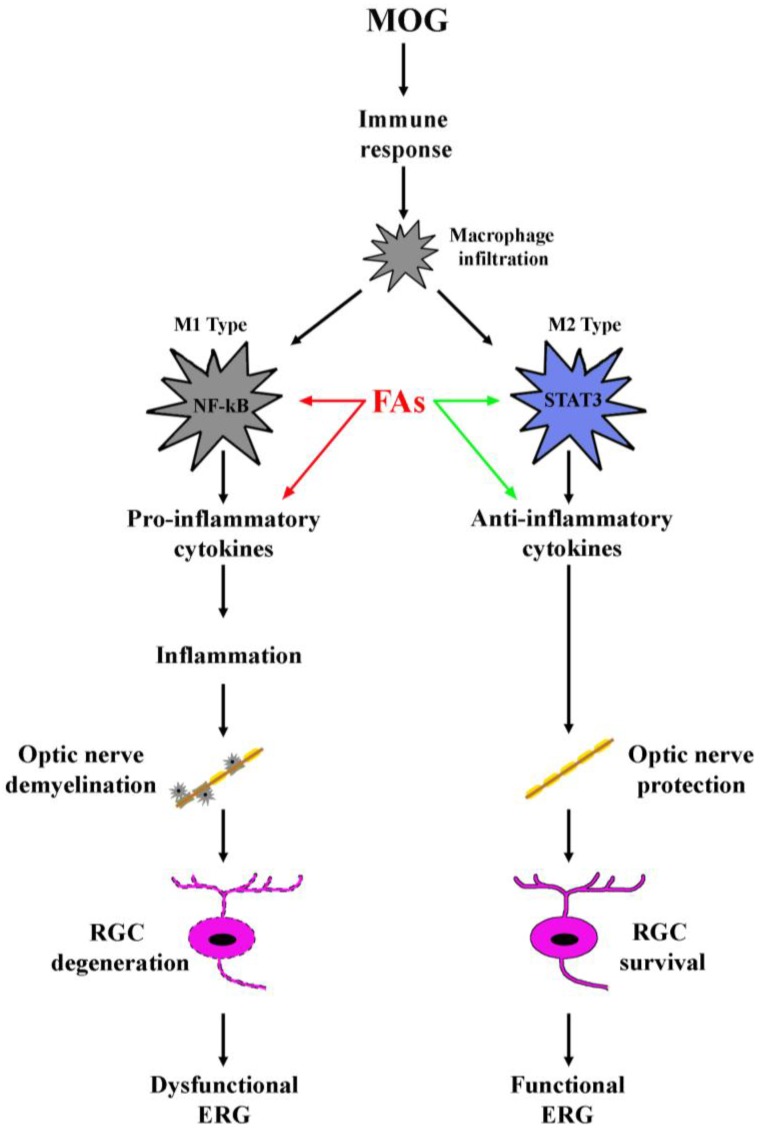
Schematic diagram depicting the role of FAs in the EAE model. MOG induces an immune response resulting in macrophage infiltration. Infiltrating macrophages can acquire distinct phenotypes M1 or M2, of which, M1 produces pro-inflammatory cytokines and activates inflammatory processes, while M2 plays a role in the blockade of inflammation and promotes tissue repair. Both FAs-induced inhibition of M1 phenotype (red arrows) and FAs-induced activation of M2 phenotype (green arrows) concur to prevent inflammatory processes in the retina thus hampering optic nerve damage and retinal ganglion cell (RGC) death, events that both participate to counteract electroretinogram (ERG) dysfunction.

As shown in the schematic representation of Figure 6, the present data demonstrate an ability of FAs to preserve significantly visual dysfunction by substantially preventing RGC loss and restoring optic nerve damage through a major anti-inflammatory action including an effect on macrophage polarization. Future studies should be planned to establish the necessary doses of FAs needed to achieve significant beneficial effects as well as to encourage the development and consumption of foods and/or supplements rich in FAs.

## Supplementary Materials

The following are available online at http://www.mdpi.com/2072-6643/10/10/1447/s1, Table S1: Sequences of primer sets used for qPCR experiments. Table S2: List of the antibodies used in Western blot experiments.
