# Supplementary material for: Further Evidence on Efficacy of Diet Supplementation with Fatty Acids in Ocular Pathologies: Insights from the EAE Model of Optic Neuritis
Source: Nutrients. 2018 Oct 6;10(10):1447. doi: 10.3390/nu10101447 (PMC6213612; doi:10.3390/nu10101447)
Supplement: Supplementary file 1 [file nutrients-10-01447-s001.pdf]

Table S1. Sequences of primer sets used for qPCR experiments.

Gene	Primer sequence (5' –> 3')	
	**Forward Primer**	**Reverse Primer**
CXCL-10	CCAAGTGCTGCCGTCATTTT	CTCAACACGTGGGCAGGATA
CXCL-11	GCTGCTCAAGGCTTCCTTATGT	ACTTTGTCGCAGCCGTTACT
IL-12	GTGGAATGGCGTCTCTGTCT	CGGGTCTGGTTTGATGATGT
IL-23	CCCGTATCCAGTGTGAAGATG	AGGGAGGTGTGAAGTTGCTC
CCL-2	ACAAGAGGATCACCAGCAGC	ATTCCTTCTTGGGGTCAGCAC
CCL-22	GCTGTGGCAATTCAGACCTC	TGACGGATGTAGTCCTGGCA
CD-163	ATGCTTCCATCCAGTGCCTC	CACAAACCAAGAGTGCCGTG
Arg-1	CAGCACTGAGGAAAGCTGGT	CAGACCGTGGGTTCTTCACA
Rpl13a	CACTCTGGAGGAGAAACGGAAGG	GCAGGCATGAGGCAAACAGTC

Table S2. List of Antibodies using in Wester blot.

**Antibody**	**Dilution**	**Source**	**Cat. No.**
Mouse monoclonal anti-β-actin	1:2500	Sigma-Aldrich Corp	A2228
Rabbit polyclonal anti-STAT3	1:200	Santa Cruz Biotechnology	sc-482
Mouse monoclonal anti-pSTAT3 (Tyr^{705})	1:200	Santa Cruz Biotechnology	sc-8059

Rabbit policlonal anti-NF-kB p65	1:200	Santa Cruz Biotechnology	sc-372
Rabbit policlonal anti-pNF-kB p65 (Ser$_{276}$)	1:200	Santa Cruz Biotechnology	sc-101749
Rabbit policlonal anti-Arg-1	1:100	ThermoFisher Scientific Inc	pa5-32267
Goat policlonal anti-IL-10	1:100	Santa Cruz Biotechnology	sc-1783
